# Multiple ovulation and embryo transfer in sheep: Effects of embryo developmental stage and quality on viability in vivo under farm conditions

**DOI:** 10.1111/avj.13174

**Published:** 2022-05-18

**Authors:** CAF King, D Osborn, CG Grupen

**Affiliations:** ^1^ Sydney School of Veterinary Science, Faculty of Science The University of Sydney Camden New South Wales 2570 Australia; ^2^ Apiam Genetic Services Dubbo New South Wales 2830 Australia

**Keywords:** embryo transfer, embryonic development, ovine, superovulation

## Abstract

Multiple ovulation and embryo transfer (MOET) technologies are integral to genetic improvement programs in the sheep industries. Despite the protocols being well established, previous findings regarding the effects of embryo properties on MOET success remain contradictory. The objective of this study was to determine the effects of embryo developmental stage and quality on embryo viability following transfer to recipient ewes. Data including details of 377 embryos collected from 45 Merino donor ewes were obtained from MOET trials conducted on three separate farms on day 6 after laparoscopic artificial insemination (AI). A total of 270 embryos were classified as being of transferrable grade (grade 1: n = 233; grade 2: n = 37). One or two transferrable grade embryos were transferred to each of 256 synchronised recipient ewes and pregnancy diagnosis was performed on day 36 after embryo transfer. Embryos at the hatched blastocyst stage tended to have greater viability in vivo compared to embryos at the late morula stage (59.0 ± 10.6% vs. 36.2 ± 9.7%; P = 0.083). The viability of grade 1 embryos was greater than that of grade 2 embryos (53.6 ± 7.8% vs. 35.9 ± 10.2%; P < 0.05). The results suggest that the success of the MOET trials was influenced by the transfer of embryos at the late morula stage, almost half of which were classified as grade 2 embryos. These findings highlight the importance of following strict embryo quality grading criteria to inform the most economical management of recipient ewes and maximize pregnancy outcomes.

Multiple ovulation and embryo transfer (MOET) technologies are an integral part of breeding schemes in the sheep industries, enabling a rapid increase in the numbers of high genetic merit animals.^1^, ^2^, ^3^, ^4^ For these technologies to be efficiently implemented, the number of progeny born from genetically superior females must be maximised.^5^, ^6^ Whilst affected by numerous factors, the number of lambs born per donor female are directly related to the viability of transferred embryos.^5^ Embryo viability in various species has been found to be affected by embryo developmental stage and quality at the time of transfer, the number of embryos transferred and the type of semen (fresh or frozen‐thawed) used for artificial insemination (AI).^6^, ^7^

There is a lack of scientific consensus regarding the effect of the embryo developmental stage on embryo viability following transfer. Numerous investigations in sheep and other species have reported conflicting results, with some concluding that an increase in the stage of development of embryos results in increased viability,^8^, ^9^, ^10^ and others determining that there is no effect.^5^, ^7^, ^11^, ^12^, ^13^, ^14^, ^15^ Studies in cattle investigating the effect of embryo quality on embryo viability following transfer have given varying results, with some studies describing improved pregnancy rates with increasing embryo quality.^9^ Other studies describe that whilst extreme grades of embryo quality affect viability, embryos of close quality grades do not have different survival rates.^16^ In sheep, prior research suggests that embryo quality has a significant effect on viability, with reduced survival as embryo quality declines.^5^ However, similar to the findings of cattle studies, other sheep studies indicate that there is no difference in embryo viability for embryos of similar grades.^5^, ^17^

The effect of the number of embryos transferred to a recipient female on embryo viability has been explored across a number of species. Studies report that the survival rate of embryos improved when two embryos were transferred to goats.^18^ Similarly, Sreenan and Diskin^16^ reported that in cattle, pregnancy rates increased for twin embryo transfers, compared with a single embryo transfer. Moore, Rowson and Short^19^ suggested that the embryo survival rate in ewes receiving five embryos was significantly lower compared with ewes that received only two embryos. In addition, the type of semen (fresh or frozen‐thawed) used for AI has the potential to affect pregnancy rate, as cryopreservation can cause significant damage to sperm cells.^20^, ^21^ Some previous sheep studies have found that the use of frozen‐thawed semen reduced embryo viability, while others conclude that semen type had no effect on embryo viability after transfer.^6^, ^22^

The objective of this study was to evaluate the effects of the stage of embryo development and embryo quality (grades 1 and 2) at the time of collection (on day 6 after AI), on embryo viability following transfer to recipient ewes. In addition, this study examined the effects of the number of embryos transferred (one or two), semen type (fresh or frozen‐thawed) used for AI and trial site (three farms) on embryo viability in vivo.

## Materials and methods

### Farms and general procedures

Data were obtained from three MOET trials performed on‐farm in Autumn (March to April), at three different properties (hereinafter referred to as farms A, B and C) in the Central West and Hunter regions of New South Wales, Australia. Superovulation of donor ewes and synchronisation of recipient ewes were performed according to standard protocols (Table A1). All laparoscopic AIs, laparoscopic embryo collections from donor ewes and embryo transfers to recipient ewes were performed by a sheep reproduction veterinarian with extensive experience (D. Osborn). All procedures were carried out according to the guidelines of the Australian Code of Practice for the Use of Animals for Scientific Purposes.^23^

### Animals

A total of 45 Merino ewes were programmed as embryo donors and 256 Merino ewes were used as embryo recipients (farm A: 12 donors and 65 recipients; farm B: 23 donors and 140 recipients; farm C: 10 donors and 51 recipients). The animals were selected from commercial breeding flocks by the managers of the three farms. A total of 377 embryos were recovered from the donor ewes on day 6 after laparoscopic AI and 270 of these embryos were classified as being of transferrable grade.

### Superovulation and synchronisation protocols

For superovulation, the donor ewes received an intravaginal controlled internal drug release (CIDR) device containing 0.3 g of progesterone (Zoetis, Florham Park, NJ, USA) on the morning of day 0 of treatment (Table A1). On day 7 of treatment, the CIDR devices were replaced and the ewes received 0.25 mg of prostaglandin F2α (PGF2α) analogue IM (cloprostenol; Ovuprost; Bayer Australia Ltd, Pymble, NSW, Aust). From day 11 of treatment, ewes received a total of 250 mg of follicle stimulating hormone (Folltropin‐V; 20 mg/mL; Vetoquinol Australia, Hamilton, QLD, Aust), administered as 8 injections IM (50, 40, 40, 40, 30, 20, 20 and 10 mg) over a 4 days period. On day 13 of treatment, the CIDR devices were removed and the ewes received 400 IU of equine chorionic gonadotropin (eCG) (Pregnecol; Vetoquinol Australia, QLD, Aust).

For oestrus synchronisation, the recipient ewes were implanted with an intravaginal CIDR device on the morning of day 0 of treatment (Table A1). On the morning of day 13 of treatment, the CIDR devices were removed and the ewes were injected with 600 IU of eCG IM.

### Collection and preparation of semen

The semen used in this study was collected from 15 rams selected by the managers of the three farms. All sperm parameters were within the normal range for ram semen (volume: 0.75–2 mL; sperm concentration: ≥3.2 × 10^9^ spermatozoa/mL; sperm motility: ≥70%; normal sperm morphology: ≥90%).

The freshly collected semen to be used as fresh semen was diluted with a warmed OviPlus extender (Minitube Australia, Smythesdale, VIC, Aust) to a dilution ratio of 200 × 10^6^ spermatozoa/mL and maintained at 30°C until insemination (within 1 h of collection).

For frozen‐thawed semen, the freshly collected semen was diluted with a Tris‐based extender (Triladyl; Minitube Australia) to a dilution ratio of 200 × 10^6^ spermatozoa/mL at room temperature. The semen was then gradually cooled over a period of 90 min to 4°C. The chilled semen was then packaged into 0.25 mL French straws. The straws were subsequently held 4 cm above liquid nitrogen for 10 min and then immersed and stored in liquid nitrogen. Immediately prior to use, the semen was thawed by immersing the straws in a water bath at 37°C for 30 s. The thawed semen was then placed into warmed 10 mL glass tubes and 0.01 mg/mL of sperm dye (Minitube Australia) was added. The post‐thaw motility of frozen‐thawed semen was evaluated by phase‐contrast microscopy. All frozen‐thawed semen used for laparoscopic AI had post‐thaw progressive motility greater than 40%. After evaluation, the frozen‐thawed semen was held at 30°C until insemination (within 20 min of thawing).

### Laparoscopic AI

When fresh semen was used, AI of embryo donor ewes was conducted 38–39 h after CIDR removal. When frozen‐thawed semen was used, AI of embryo donor ewes was conducted 39–40 h after CIDR removal and repeated approximately 6 h later. The ewes were fasted for at least 20 h prior to AI. In preparation for the procedure, each ewe was sedated with xylazine (ilium Xylazil‐20; 0.08–0.12 mg/kg; Troy Laboratories Pty Ltd, Glendenning, NSW, Aust) and placed head‐down in dorsal recumbency at a 45° angle in a laparoscopic cradle. The belly wool was clipped and a chlorhexidine surgical scrub (4% wt/vol chlorhexidine gluconate, Schulke Australia Pty Ltd, Macquarie Park, NSW, Aust) was used to clean the lower abdominal area, which was subsequently rinsed thoroughly and dried using gauze sponges. Two small incisions were made on either side of the abdominal midline, approximately 8–10 cm anterior to the mammary glands, for insertion of the laparoscope and insemination pipette via cannulas. A pneumoperitoneum was produced by insufflating the abdominal cavity with carbon dioxide gas via the laparoscopic cannula. Intrauterine insemination was carried out using freshly collected semen (farms A and B) or frozen‐thawed semen (farms B and C). For each ewe, the prepared semen was loaded into a Robertson pipette (Minitube Australia; 0.2 mL containing a total of 25 × 10^6^ progressively motile spermatozoa), with half (0.1 mL) of the insemination dose deposited into the lumen at the middle of each uterine horn. After the procedure, the ewes were removed from the cradles, allowed to recover in a nearby pen and then returned to pasture.

### Embryo collection

Embryos were recovered on day 6 after AI using a laparoscopic procedure. The ewes were fasted for at least 20 h prior to embryo collection. In preparation for the procedure, each ewe was sedated with xylazine (0.08–0.12 mg/kg) and placed head‐down at a 45° angle in a laparoscopic cradle. Chlorhexidine 4% surgical scrub was used to clean the lower abdominal area, which was subsequently rinsed thoroughly and dried using gauze sponges. Inhalation anaesthesia was induced using a mix of isoflurane, nitrous oxide and oxygen. An abdominal incision approximately 7 cm in length was made and the uterine horns were externalised. Subsequently, a hole was made near the uterine bifurcation using a blunt 19‐G needle. A 10‐G Foley catheter (Pacific Vet, Braeside, VIC, Aust) was passed through this hole and positioned into the lumen of the proximal third of one of the uterine horns. Twenty millilitres of flushing medium (Vigro complete flush; Minitube Australia) warmed to room temperature (25–30°C) was used to flush the uterine contents into a collection dish. This flushing procedure was repeated for the second uterine horn with the uterine contents collected into a separate dish. The flushings were maintained at room temperature during embryo searching and assessment. Finally, the uterine horns were repositioned, the instruments removed and the abdominal incision sutured. After the procedure, the ewes were removed from the cradles, allowed to recover in a nearby pen and then returned to pasture.

### Embryo assessments

The recovered embryos and ova were assessed immediately by light microscopy and each was classified as either an unfertilised ovum (UF), degenerate or arrested embryo (DG), late morula (LM), early blastocyst (EB), blastocyst (BL), expanded blastocyst (XB), or hatching blastocyst (HB). All embryos at the late morula to hatching blastocyst stages were further categorised into quality grades (1 = excellent, 2 = good, 3 = poor). The morphological scoring of embryo quality was based on criteria according to International Embryo Technology Society (IETS) conventions.^24^ Only grade 1 and 2 embryos were determined to be of transferrable grade. The embryos were washed and kept in an embryo‐holding medium (BoviHold; Minitube Australia) at room temperature until transfer.

### Embryo transfer

Transferrable grade embryos were immediately transferred to recipient ewes using a laparoscopic procedure. The ewes were fasted for at least 20 h prior to embryo transfer. Each ewe was prepared for the procedure as described for laparoscopic AI and inhalation anaesthesia was induced using a mix of isoflurane, nitrous oxide and oxygen. The ovaries were examined to confirm the presence of at least one normal corpus luteum (CL). If a CL was not found on either ovary, the ewe was not used. Uterine grasping forceps were used to exteriorise the tip of the uterine horn ipsilateral to the CL‐bearing ovary. One or two embryos were loaded into a Tomcat embryo transfer catheter (Minitube Australia) with a minimal volume of medium bracketed by small air bubbles. Single or double embryos were deposited into the uterine lumen, approximately 2 cm from the utero‐tubal junction via a hole made using a blunt 19‐G needle. Finally, the exteriorised uterine horn was repositioned, the instruments removed and the incisions sutured.

### Assessment of embryo viability in vivo

Ultrasonographic scanning by commercial ultrasound technicians was performed to determine the pregnancy status and number of foetuses for each recipient ewe at 36 days after embryo transfer. All pregnancies were permitted to be carried to term.

### Statistical analyses

The fertilisation and transferrable grade embryo results were subjected to analysis of variance with farm and semen type as factors. The distributions of embryos categorised according to developmental stage and quality were analysed using chi‐square tests. The effects of embryo developmental stage and quality at the time of collection on embryo viability in vivo were analysed using a logistic generalised linear mixed model (GLMM) with stage and grade as factors. The outcome of the in vivo viability analysis was pregnancy status (1 = pregnant, 0 = not pregnant). The effects of the farm, semen type and the number of embryos transferred per recipient ewe on embryo viability in vivo were analysed using a GLMM with the farm, semen type and number transferred as factors. Random effects to account for clustering were sire, donor and recipient. The model was fitted using ASReml‐R^25^ using the statistical package R. A P value of less than 0.05 designated a significant difference.

## Results

### Embryo collections

The 45 donor ewes programmed to superovulate all responded to treatment and all were flushed. The results of the embryo collections are shown in Table 1. A total of 377 structures (ova and embryos) were recovered, of which 339 were embryos. Of these, 305 were determined to be of transferrable grade, giving a mean of 6.78 transferrable grade embryos collected per donor ewe. The proportions of ova that were fertilised and the proportions of embryos considered to be of transferrable grade did not differ significantly between the farms (Table 1; P > 0.05). Also, the type of semen used for AI did not affect the proportions of ova that were fertilised (95.0 ± 5.4% vs. 79.2 ± 6.9%; P > 0.05) or the proportions of embryos considered to be of transferrable grade (86.5 ± 4.4% vs. 95.1 ± 6.0%; P > 0.05).

**TABLE 1:** Summary of the embryo collections at the three farms

Farm	Donor ewes	Embryos/ova recovered	Ova fertilised (%^a^)	Transferrable embryos (%^a^)	Transferrable embryos/donor
A	12	139	91.5 ± 7.8	88.9 ± 6.7	9.08
B	23	157	94.1 ± 5.9	89.6 ± 5.1	6.26
C	10	81	70.9 ± 9.8	90.1 ± 8.8	5.20
Total	45	377	87.0 ± 13.8	89.7 ± 3.8	6.78

^a^ Values are expressed as the mean ± SE.

For the analyses of in vivo viability, 35 of the 305 transferred embryos were excluded from the data as their viability could not be determined definitively by ultrasonographic scanning, resulting in a total of 270 embryos. Most of these embryos were transferred as singles to 242 recipient ewes and 28 embryos were transferred as doubles to 14 recipient ewes. Of the 270 embryos with a definitive pregnancy diagnosis, there were 233 embryos classed as grade 1 and 37 classed as grade 2.

### Distributions of embryo developmental stages and grades

**Figure 1:**
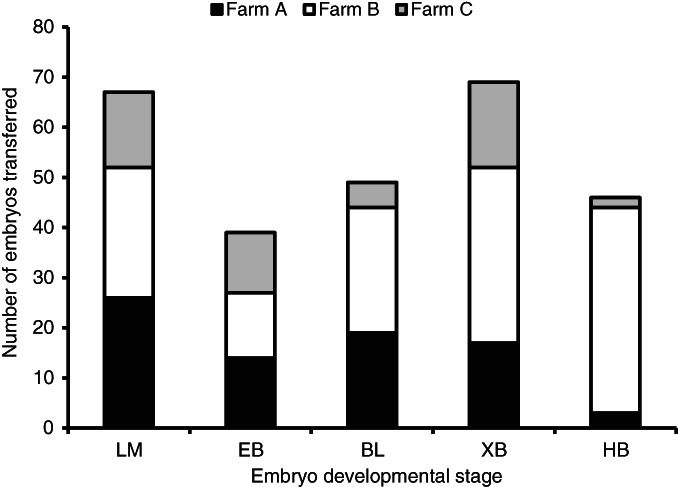
The distribution of transferred embryos collected on day 6 after AI at farms A, B and C according to the morphological assessment of developmental stage at the time of collection. BL, blastocyst; EB, early blastocyst; HB, hatching/hatched blastocyst; LM, late morula; XB, expanded blastocyst.

At the time of collection, the embryos transferred at farms A, B and C varied from the late morula to the hatched blastocyst stages of development (Figure 1). At farm B, the proportion of embryos at the hatching blastocyst stage was greater than at farms A and C (P < 0.05). Concurrently, smaller proportions of embryos at farm B were at the late morula and early blastocyst stages compared with farms A and C, respectively (P < 0.05). The proportions of embryos at each developmental stage were similar at farms A and C except for the blastocyst stage, which was greater at farm A (P < 0.05).

**Figure 2:**
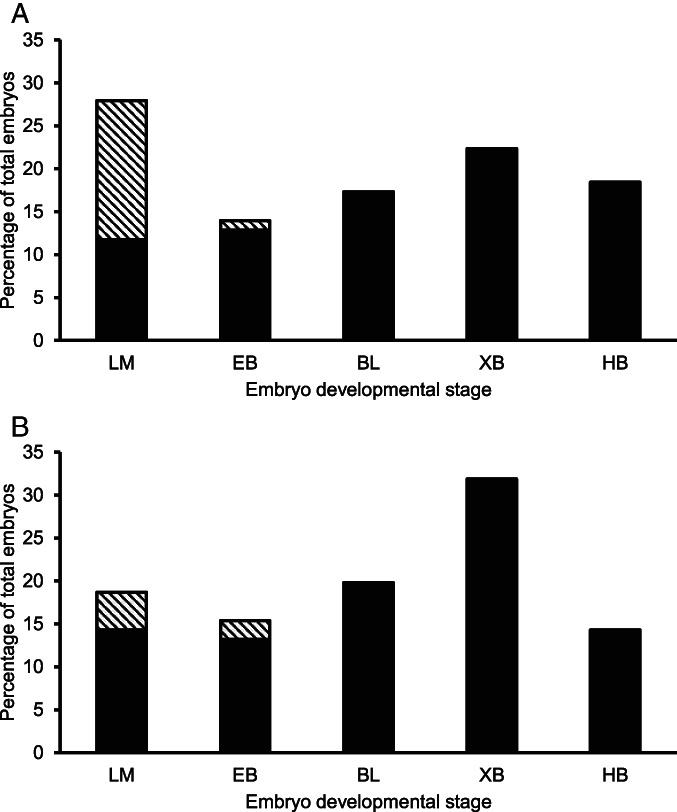
The distribution of transferred embryos derived from (A) fresh and (B) frozen‐thawed semen according to the developmental stage (BL, blastocyst; EB, early blastocyst; HB, hatching/hatched blastocyst; LM, late morula; XB, expanded blastocyst) and the morphological assessment of quality (grades 1 and 2 are represented by solid and hatched bars, respectively) at the time of collection.

The distribution of embryos evaluated as being of ‘excellent’ (grade 1) or ‘good’ (grade 2) quality differed according to the stage of embryo development (Figure 2). A significantly greater percentage of embryos were classified as grade 2 at the late morula stage compared with the early blastocyst stage (49.3% vs. 10.3%; P < 0.05). In turn, the percentage of embryos classified as grade 2 at the early blastocyst stage was greater than at any of the later developmental stages (10.3% vs. 0%; P < 0.05). The distribution of embryo developmental stages was similar for both fresh and frozen‐thawed semen (Figure 2; P > 0.05). Irrespective of embryo grade, the proportions of embryos at each developmental stage that were derived from fresh and frozen‐thawed semen were not significantly different (P > 0.05).

### Effects of embryo developmental stage and grade on embryo viability in vivo

The effects of embryo developmental stage and grade at the time of embryo transfer on the in vivo viability rate at day 36 after the embryo transfer are shown in Table 2. The proportions of embryos at each developmental stage found to be viable at pregnancy diagnosis did not differ significantly. However, embryos at the late morula stage tended to have a lower viability rate than those at the hatching blastocyst stage (P = 0.083). The viability rate of grade 1 embryos (53.6 ± 7.8%) was greater than that of grade 2 embryos (35.9 ± 10.2%; P = 0.026).

**Table 2:** The effects of embryo developmental stage and grade at the time of collection on the in vivo viability rate (mean ± SE) at day 36 after embryo transfer

Factor	Category	n	Viability rate (%)
Stage	Late morula (LM)	67	36.2 ± 9.7*
	Early blastocyst (EB)	39	52.8 ± 10.5
	Blastocyst (BL)	49	58.1 ± 10.1
	Expanded blastocyst (XB)	69	52.3 ± 9.5
	Hatched blastocyst (HB)	46	59.0 ± 10.6*
Grade	Excellent (1)	233	53.6 ± 7.8^a^
	Good (2)	37	35.9 ± 10.2^b^

Values labelled with different letters are significantly different (P = 0.026). Values labelled with an asterisk tend to differ (P = 0.083).

### Effect of the farm on embryo viability in vivo

**FIGURE 3:**
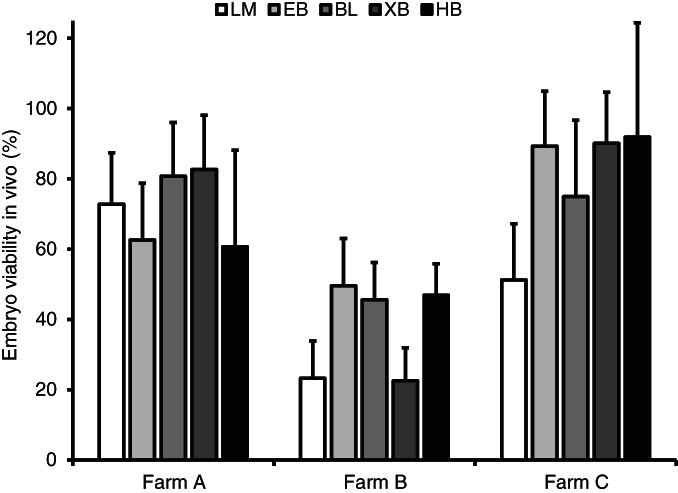
The effect of the farm on the in vivo viability rate (mean ± SE) at day 36 after embryo transfer shown for each developmental stage at the time of collection. BL, blastocyst; EB, early blastocyst; HB, hatching/hatched blastocyst; LM, late morula; XB, expanded blastocyst.

The effect of the farm on the in vivo viability rate at day 36 after embryo transfer is shown in Table 3. Embryos collected and transferred at farm B had significantly lower viability in vivo compared with those at farms A and C (33.3 ± 6.8% vs. 76.2 ± 10.7% and 77.9 ± 11.8%, respectively; P = 0.016). Whilst the analysis showed there was no significant interaction between the factors of farm and embryo developmental stage at the time of collection, the in vivo viability of embryos at the late morula and expanded blastocyst stages appeared to be markedly reduced at farm B (Figure 3).

**Table 3:** The effects of the farm, the semen type used for artificial insemination and the number of embryos transferred to each recipient ewe on the in vivo viability rate (mean ± SE) at day 36 after embryo transfer

Factor	Category	n	Viability rate (%)
Farm	A	79	76.2 ± 10.7^a^
	B	140	33.3 ± 6.8^b^
	C	51	77.9 ± 11.8^a^
Semen type	Fresh	179	49.2 ± 7.5
	Frozen–thawed	91	58.6 ± 10.1
Number transferred	Single	242	50.8 ± 7.7
	Double	28	51.6 ± 12.3

Values labelled with different letters are significantly different (P = 0.016).

### Effect of semen type on embryo viability in vivo

The effect of the semen type used for AI (fresh or frozen‐thawed) on the in vivo viability rate at day 36 after embryo transfer is shown in Table 3. The viability rate of embryos produced using frozen‐thawed semen did not differ significantly from that of embryos produced using fresh semen (58.6 ± 10.1% vs. 49.2 ± 7.5%; P = 0.457).

### Effect of the number of embryos transferred on embryo viability in vivo

The effect of the number of embryos transferred to each recipient ewe on the in vivo viability rate at day 36 after embryo transfer is shown in Table 3. The viability rate of embryos transferred as singles was nearly identical to that of embryos transferred as doubles (50.8 ± 7.7% vs. 51.6 ± 12.3%; P = 0.936).

## Discussion

The results of this study show that the embryo developmental stage at the time of collection on day 6 after AI did not affect embryo viability in vivo. Whilst not significant, the in vivo viability of embryos transferred at the morula stage tended to be lower than that of embryos transferred at the more advanced stages of development. However, there was a difference in viability in vivo between embryo quality grades as assessed by morphological criteria. As most of the poorer quality embryos were at the morula stage of development at the time of collection, this may explain the observed tendency for the embryo developmental stage to influence subsequent viability. Previous MOET studies in both sheep and cattle provide inconsistent findings regarding the effect of embryo developmental stage on pregnancy outcomes.^5^, ^8^, ^9^, ^10^

Alternatively, the observed results may be attributed to the relationship between the embryo stage and the uterine environment. In the present study, embryos were collected on day 6 after AI, when the recovered embryos would be expected to be at the blastocyst stage of development. As found previously in sheep, the spread of embryo developmental stages was considerable, from the late morula to the hatching blastocyst stages. Embryos at the blastocyst stage displayed higher viability in vivo, possibly due to their high synchrony with the uterine environment of the recipient ewes.^13^ Conversely, embryos at the late morula stage, which is somewhat delayed in development at day 6 after AI, may have had poorer viability in vivo due to their asynchrony with the uterine environment.^5^ Interestingly, embryos transferred at the hatching blastocyst stage, which is somewhat advanced in development, did not display poorer viability in vivo. Previous studies in sheep and pigs have found that synchronously retarded embryos are much less likely to survive following implantation than synchronous or synchronously advanced embryos.^13^, ^15^, ^26^ Given that the effect of embryo developmental stage was not significant in the small number of MOET trials examined here, larger‐scale studies may be needed to detect differences.

Embryos morphologically assessed as being of ‘excellent’ quality (grade 1), according to IETS conventions, had a greater rate of viability in vivo than those assessed as being of ‘good’ quality (grade 2). Previous studies in cattle and sheep have shown that pregnancy rates improve with increasing embryo quality.^5^, ^9^, ^16^ While embryos of extreme quality grades would be expected to differ in viability, there are some reports where embryos of close quality grades did not differ significantly in post‐transfer survival rates.^5^, ^16^, ^17^, ^28^ Although the morphological evaluation of embryo grade is based on well‐defined IETS criteria,^24^ the assignment of embryos to a particular grade is subjective.^5^, ^16^, ^27^ Therefore, differentiation between close quality grades may be difficult and prone to subjective bias.^16^, ^27^ In the present study, all of the embryo assessments were performed by the one highly experienced sheep reproduction specialist, which ensured consistency across the trials. A feature of these evaluations was that most of the grade 2 embryos were at the morula stage and nearly half of the embryos at the morula stage were of grade 2 quality (Figure 2). The other grade 2 embryos were at the early blastocyst stage and none of the embryos at the more developed blastocyst stages were of grade 2 quality. To our knowledge, the distribution of embryo quality grades according to embryo developmental stage has not been reported previously. It has been proposed that the reduced survival of inferior quality embryos is due to their lower tolerance to an asynchronous uterine environment.^16^ We suggest that such embryos are often transferred in practice, reducing the overall efficiency of MOET programs. These findings highlight the importance of following strict morphological criteria to determine embryo quality.

There was no difference in embryo viability in vivo when one or two embryos were transferred to recipient ewes. This result is consistent with a previous study conducted in sheep that reported similar embryo viabilities in ewes that received one or two embryos.^8^ However, other studies conducted in goats and cattle have reported an increase in embryo survival rate when two embryos were transferred.^16^, ^18^ It has previously been established that the viability of twin embryos is significantly greater when both embryos are transferred to the same oviduct (unilateral transfer) compared to when one embryo is transferred to each oviduct (bilateral transfer).^18^ As most reports do not describe whether twin embryos were transferred unilaterally or bilaterally, the discrepancy between findings may be due to the transfer method used. While Moore, Rowson and Short^19^ concluded that the proportion of ewes becoming pregnant was the same when either five or two embryos were transferred, the individual embryo survival rate in ewes that received five embryos was lower than that in ewes that received only two embryos. It is proposed that the embryo survival rate is dependent on the ability of the recipient ewe to support a pregnancy, rather than on the number of embryos transferred.^19^ Further research is needed to clarify the effect of the number of embryos transferred on embryo viability following transfer.

The results of the present study indicate that the type of semen used for AI did not affect the in vivo viability of the resulting embryos. It is well established that the freeze‐thaw process can cause significant cellular and DNA damage, compromising the fertilising ability of ram spermatozoa.^20^, ^21^, ^28^, ^29^ While some early intrauterine AI studies in ewes found that fertilisation and pregnancy rates were lower for frozen‐thawed semen than for fresh semen,^29^, ^30^ the laparoscopic AI method is regarded as reliable, with both fresh and frozen‐thawed semen achieving commercially acceptable fertilisation rates in superovulated ewes.^6^, ^22^ In order to maximise the effectiveness of frozen‐thawed semen in MOET trials, depositing the semen approximately 6–10 h prior to expected ovulation, as well as performing double insemination, is recommended.^21^ In the present study, the MOET trials that utilised frozen semen (farms B and C) had AI conducted within this time period prior to expected ovulation and double insemination was performed. There were no effects of semen type (fresh or frozen‐thawed) on the distribution of transferred embryos according to the morphological assessment of quality and developmental stage. Furthermore, there were no effects of semen type on the proportions of ova that were fertilised, or the proportions of embryos classified as being of transferrable grade.

There was a clear difference between embryo viability in vivo between farms, with the viability rate at farm B being significantly lower than at farms A and C. Also, the mean number of transferrable grade embryos collected per donor ewe appeared to be greater at farm A than at farms B and C. As the one highly skilled operator performed all the AI and embryo procedures at the three farms, the differences were not due to technique. Other factors that could not be controlled at the commercial enterprises may have contributed to the observed differences. For example, the ewe hormone treatments were administered by the staff at each farm, which may have introduced some variance in the timing they were given. Such variance may result in small deviations in the superovulatory response and the expected time of ovulation in donor ewes. However, the embryo collection results did not differ significantly between the farms, indicating that any discrepancies in the hormone treatments were minor. Ewe factors, such as maternal age, body condition and physiological status, are known to influence the ovulatory response and embryo quality.^5^, ^7^, ^31^, ^32^ Whilst all donor and recipient ewes selected for the trials were of good body condition (Body Condition Score of 3 to 4), the other ewe factors were not standardised. Seasonal and environmental effects can be largely disregarded, as the trials were all carried out within a three‐week period during the breeding season. Another variation between the farms was the use of fresh and frozen‐thawed semen, with farm A using fresh only, farm B using fresh and frozen‐thawed and farm C using frozen‐thawed only. As the poorest viability rate was achieved at farm B, this suggests that semen type had no effect on embryo viability in vivo and suggests the farm effect was related to ewe factors or management practices.

Originally, all the transferrable grade embryos were planned to be transferred as single embryos, but some of the recipient ewes were deemed unsuitable because they lacked a normal corpus luteum on either ovary. To maximise the pregnancy outcomes for the farms, some of the embryos were consequently transferred as doubles. Whilst this enabled the comparison of single and double embryo transfers to be made, the analysis was limited due to the small number of recipients that received two embryos. Also, the pregnancy diagnosis was more complicated for ewes carrying two embryos, which accounted for most of these embryos being excluded from the analysis of in vivo viability. It should be noted that the ultrasound scanning was performed by different technicians as a commercial service at each farm. Whilst the accuracy of pregnancy diagnosis was expected to be very high, slight differences between operators may be another potential source of variance. Monitoring the pregnancies to lambing would have been ideal to gain information on the full‐term developmental capacity of the embryos. Although these issues highlight the limitations of carrying out studies on commercial farms, such trials demonstrate the robustness of the MOET procedures under real‐world farm conditions and provide data that is perhaps of even more significance to the sheep industry.

## Conclusion

Our results show that embryo quality, as evaluated using IETS conventions, had the greatest effect on embryo viability in vivo. The spread of development in embryos from the late morula to the hatching blastocyst stages collected on day 6 after AI is considerable in sheep, but despite the asynchrony with the uterine environment of the recipient ewe, only late morula stage embryos tended to have lower viability in vivo compared with later‐stage embryos. To the best of our knowledge, this is the first MOET study to report that the distribution of grade 2 embryos was skewed towards developmentally delayed embryos, which may provide a basis for the inconsistent findings of previous MOET studies. The number of embryos transferred to each recipient ewe and the semen type used for AI did not affect embryo viability. These findings highlight the importance of following strict embryo quality grading criteria to inform the most economical management of recipient ewes and maximize the efficiency of breeding programs that incorporate MOET technologies.

## Conflicts of interest and sources of funding

The authors declare no conflicts of interest or sources of funding for the work presented here.

## Appendix A

### A.1

**Table A1:** Protocols for the superovulation of donor ewes and synchronisation of recipient ewes

Day	Time	Animals	Activity	Treatment	Dose/AI timing
0	8 am	Donors Recipients	Insert CIDRs Insert CIDRs		
7	8 am	Donors	Change CIDRs and administer drug	Cloprostenol	0.25 mg IM
11	8 am 6 pm	Donors Donors	Administer drug Administer drug	FSH FSH	50 mg IM 40 mg IM
12	8 am 6 pm	Donors Donors	Administer drug Administer drug	FSH FSH	40 mg IM 40 mg IM
13	6 am	Recipients	Remove CIDRs and administer drug	eCG	600 IU IM
	8 am	Donors	Administer drug	FSH	30 mg IM
	6 pm	Donors	Remove CIDRs and administer drug	FSH eCG	20 mg IM 400 IU IM
14	8 am	Donors	Administer drug	FSH	20 mg IM
	6 pm	Donors	Administer drug	FSH	10 mg IM
			Remove water and feed		
15	8–9 am	Donors	AI (fresh semen)		38–39 h^a^
	9–10 am	Donors	AI (frozen semen)		39–40 h^a^
	3–4 pm	Donors	AI (frozen semen)		45–46 h^a^
20	1 pm	Donors	Remove water and feed		
	5 pm	Recipients	Remove water and feed		
21			Collect and transfer embryos		

^a^ Hours after CIDR removal.

AI, artificial insemination; CIDR, controlled internal drug release; eCG, equine chorionic gonadotropin; FSH, follicle stimulating hormone; IM, intramuscular.
